# Anticorrosion and Antimicrobial Tannic Acid-Functionalized Ti-Metallic Glass Ribbons for Dental Abutment

**DOI:** 10.1021/acsabm.3c00948

**Published:** 2024-02-01

**Authors:** Eray Yüce, Elham Sharifikolouei, Matej Micusik, Sara Ferraris, Reza Rashidi, Ziba Najmi, Selin Gümrükçü, Alessandro Scalia, Andrea Cochis, Lia Rimondini, Silvia Spriano, Maria Omastova, Abdulkadir Sezai Sarac, Jürgen Eckert, Baran Sarac

**Affiliations:** † Erich Schmid Institute of Materials Science, Austrian Academy of Sciences, 8700 Leoben, Austria; ‡ Department of Materials Science, Chair of Materials Physics, Montanuniversität Leoben, 8700 Leoben, Austria; § Department of Applied Science and Technology (DISAT), Politecnico di Torino (POLITO), 10129 Turin, Italy; ∥ Polymer Institute, Slovak Academy of Sciences, Dubravska cesta 9, 845 41 Bratislava, Slovakia; ⊥ POLITO BIOMed LAB, Politecnico di Torino, 10129 Torino, Italy; # Department of Health Sciences, Center for Translational Research on Autoimmune and Allergic Diseases-CAAD, Università del Piemonte Orientale UPO, 28100 Novara, Italy; ∇ Department of Chemistry, Istanbul Technical University, 34469 Istanbul, Türkiye; ○ Polymer Science and Technology, Istanbul Technical University, 34469 Istanbul, Türkiye

**Keywords:** metallic glasses, ribbons, titanium, surface functionalization, antibacterial, corrosion

## Abstract

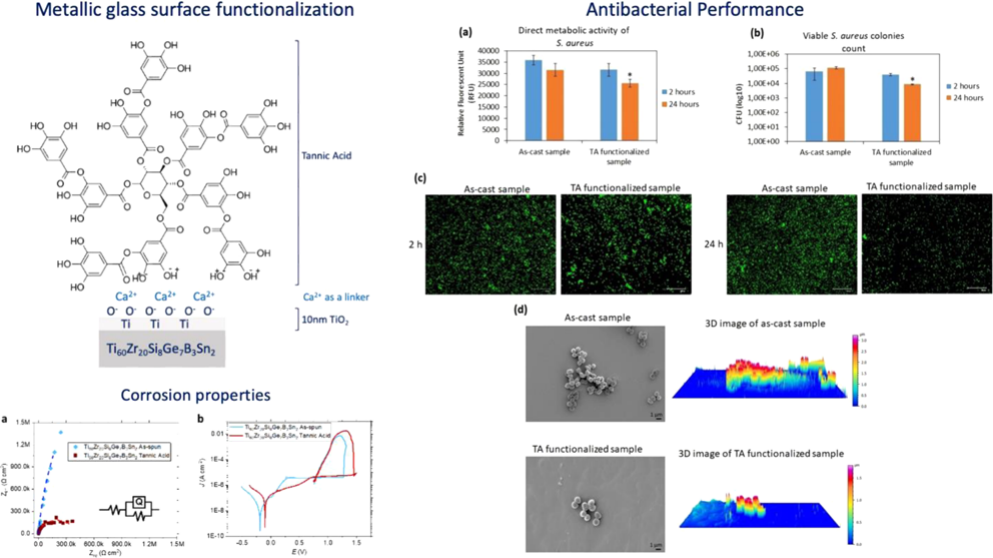

In this study, a recently reported Ti-based metallic glass (MG), without any toxic element, but with a significant amount of metalloid (Si–Ge–B, 18 atom %) and minor soft element (Sn, 2 atom %), was produced in ribbon form using conventional single-roller melt-spinning. The produced Ti_60_Zr_20_Si_8_Ge_7_B_3_Sn_2_ ribbons were investigated by differential scanning calorimetry and X-ray diffraction to confirm their amorphous structure, and their corrosion properties were further investigated by open-circuit potential and cyclic polarization tests. The ribbon’s surface was functionalized by tannic acid, a natural plant-based polyphenol, to enhance its performance in terms of corrosion prevention and antimicrobial efficacy. These properties can potentially be exploited in the premucosal parts of dental implants (abutments). The Folin and Ciocalteu test was used for the quantification of tannic acid (TA) grafted on the ribbon surface and of its redox activity. Fluorescent microscopy and ζ-potential measurements were used to confirm the presence of TA on the surfaces of the ribbons. The cytocompatibility evaluation (indirect and direct) of TA-functionalized Ti_60_Zr_20_Si_8_Ge_7_B_3_Sn_2_ MG ribbons toward primary human gingival fibroblast demonstrated that no significant differences in cell viability were detected between the functionalized and as-produced (control) MG ribbons. Finally, the antibacterial investigation of TA-functionalized samples against *Staphylococcus aureus* demonstrated the specimens’ antimicrobial properties, shown by scanning electron microscopy images after 24 h, presenting a few single colonies remaining on their surfaces. The thickness of bacterial aggregations (biofilm-like) that were formed on the surface of the as-produced samples reduced from 3.5 to 1.5 μm.

## 1 Introduction

Titanium and its alloys, such as commercially pure Ti, Ti–6Al–4V, Ti–6Al–7Nb, and Ti–5Al–2.5Fe, have gained widespread use in dental, trauma, and orthopedic surgery, thanks to their exceptional mechanical properties and acceptable biocompatibility.^1−4^ However, these materials are not without their limitations, e.g., insufficient long-term wear resistance, the potential release of toxic metallic ions during severe corrosion, stress-shielding effects on bone, and the possibility of provoking proinflammatory responses within the human body.^1,5,6^

Two-piece dental implants, consisting of an implant body and a transmucosal part (abutment) that the dental crown is mounted on, are widely used for dental restoration due to their flexibility in prosthetic angle, based on the abutment selected (angled or straight).^7^ However, a disadvantage to the two-piece dental implant can be the existence of poor sealing along the implant body-abutment interface, providing a potential site for colonization of bacterial pathogens consisting of oral pathogens like *Aggregatibacter actinomycetemcomitans*, *Porphyromonas gingivalis*, or other pathogens, for example, *Staphylococcus aureus* and *Pseudomonas aeruginosa* transferred from anterior nares or skin.^8,9^ The literature has reported the migration of bacteria into the internal part of the bone implant through such a microgap and micromovement of the implant-abutment, causing interference with the health of the preimplant tissue, inflammation, and bone loss.^10,11^ To avoid dental implant loss due to infection and inflammation response, some antibiotics like prophylaxis were prescribed to a patient immediately after dental surgery or amoxicillin 1 h before dental surgery.^12,13^ Therefore, the development of new biomaterials without containing any toxic ions and modifying their surface properties can prevent an increased number of antibiotic-resistant bacteria due to the routine consumption of antibiotics before or after surgery. Sarac et al. demonstrated that titanium-based metallic glasses (MGs) outperform the gold-standard Ti–6Al–4V when used as dental implants.^14,15^ Yet, the development of titanium-based metallic glasses devoid of potentially toxic metals is imperative to mitigate any potential inflammatory reactions within the body.^1^ In light of this, titanium-based bulk metallic glasses (BMGs) have emerged as promising implant materials due to their advantageous engineering properties such as low elastic modulus, high strength, toughness, corrosion resistance and, in some cases, even intrinsic antibacterial properties, superior to conventional crystalline Ti-alloys.^1,16−18^ Consequently, numerous Ti-based BMGs have been reported, exhibiting favorable characteristics compared to traditional crystalline Ti-based alloys.^14,19^ Nevertheless, many of these BMGs contain elements like Ni, Cu, and/or Be, which are known to be harmful to the human body due to their cytotoxicity.^1,3−5,20−22^ As a result, their inclusion limits the application of Ti-based BMGs in the medical field. Hence, the pursuit of new glass-forming alloys without potentially toxic elements becomes imperative to effectively harness Ti-based metallic glasses for implant materials.

It should be noted that the absence of Cu, Ni, or Be remarkably restrains the glass-forming ability (GFA) of fully biocompatible Ti-based MGs. These three elements lower the liquidus temperature (*T*_liq_) significantly,^23^ and in this way, conventional BMG production methods such as suction or injection casting can be realized at relatively low cooling rates (10^2^–10^3^ K/s) sufficient to impede the crystallization of the molten bulk glass-forming alloys.^24^ However, none of the unproblematic fundamental elements^1,25^ significantly reduces *T*_liq_ as Cu, Ni, or Be does.^26^ This requires other quenching methods with much higher cooling rates (10^5^–10^6^ K/s) like melt-spinning to obtain an amorphous structure.^24,27^ Furthermore, unproblematic elements permit only metalloids (except very expensive Pd) as the best glass-forming elements since they possess the largest negative mixing enthalpies and atomic size mismatches with the main alloy constituents, Ti and Zr.

This work presents results on the surface functionalization of recently developed and completely biocompatible and corrosion-resistant Ti-based MG ribbons, namely, Ti_60_Zr_20_Si_8_Ge_7_B_3_Sn_2_.^28^ This alloy composition uses the Ti–Zr–Si-Ge pseudobinary system as a starting point. Each phase diagram of the Ti–Si, Ti–Ge, Zr–Si, and Zr–Ge binary systems has a deep eutectic reaction at 13.7 atom % of Si, 14.9 atom % of Ge, 8.8 atom % of Si, and 9.5 atom % of Ge, respectively.^26^ Likewise, for the previously reported fully biocompatible alloys, our glass-forming composition is also based on metal–metalloid-type systems. These alloys generally consist of 75–85 atom % of transition metals and 15–25 atom % of metalloids. To stabilize the glassy structure, they contain at least 15 atom % of metalloid atoms (of one type or a mixture of different metalloids) and commonly feature a deep eutectic around a composition of 15–25 atom % of metalloid. A mixture of different metalloids (Si_8_Ge_7_B_3_) was chosen to further confuse the system by increasing its entropy, while a synergistic improvement in corrosion properties and biocompatibility is also aimed for.^24^

The surface of biomaterials is a critical interface between implants and the body, affecting water, ions, proteins, cell adhesion, and tissue integration. Hence, surface engineering is vital to implant success. Surface modifications come in two main types: topographical texturing and functionalization. Laser surface texturing of titanium implants can affect surface wettability, which leads to better integration of the implant and bone growth.^29,30^ Functionalization involves the chemical modification of biomaterials by attaching active compounds to their surfaces. Unlike coatings, it combines a tailored surface texture with chemical modification but has limited capacity for binding active compounds. Compounds for functionalization are chosen based on specific goals like antibacterial action or promoting osseointegration. The type of chemical bonds formed depends on the substrate and compound characteristics. Covalent bonds offer stability but limit release, while physisorption lacks reproducibility. Chemisorption allows a controlled and pH-dependent release that is beneficial for targeted effects.^31^ In the present study, we used tannic acid polyphenols from a natural extract for functionalization, as they possess multifunctional properties,^32^ for example, antibacterial, anti-inflammatory, antitumor, antiviral, and cytocompatible characterizations that were proved thanks to the presence of high amounts of phenolic compounds and pyrogallol groups.^33^ Therefore, there has been increased interest in the implementation of tannic acid to chemically modify the surface of materials in terms of their application as implant biomaterials. A neutral pH and the addition of calcium ions to the solution used for functionalization were selected to allow the chemisorption of tannic acid onto the substrate. Calcium ions form TA–calcium complexes in the solution, and then they are expected to be electrostatically attracted by the negatively charged BMG surface. Additionally, TA can improve the corrosion resistance of the material, especially at low pH (saliva pH = 5.6–6.7) by a strong bond with Ca^2+^ ions, which are used as a linker, forming a protective layer of chelate organic compounds on the BMG surface.^34^

In this study, we present a comprehensive investigation of a recently developed Ti-based metallic glass (Ti_60_Zr_20_Si_8_Ge_7_B_3_Sn_2_), specifically tailored for dental implant applications with a focus on its biocompatibility and corrosion resistance. This innovative alloy, free from toxic elements and rich in metalloids and soft elements, was fabricated into ribbons using the single-roller melt-spinning technique. Our research encompasses a thorough analysis of the ribbons’ amorphous and chemical structure through differential scanning calorimetry (DSC), X-ray photoelectron spectroscopy (XPS), and X-ray diffraction (XRD), alongside an evaluation of their corrosion properties via open-circuit potential (OCP) and cyclic polarization (CP) tests. A key innovation in our approach is the surface functionalization of these ribbons with tannic acid, a natural polyphenol, aimed at enhancing their antimicrobial efficacy and corrosion resistance, crucial for their use in the sensitive area of dental implants. This functionalization process was verified using a range of analytical techniques, including fluorescent microscopy, scanning electron microscopy (SEM), ζ-potential measurements, and the Folin and Ciocalteu test. Our study explores the integration of natural polyphenols with metallic glasses, potentially enhancing their safety and efficacy as dental implant materials.

## 2 Materials and Methods

### 2.1 Metallic Glass Ribbon Fabrication and Characterization

A master alloy with nominal composition Ti_60_Zr_20_Si_8_Ge_7_B_3_Sn_2_ (in atom %) was prepared by arc melting (AM/0,5—Edmund Bühler) elemental Ti (99.99%), Zr (99.95%), Si (99.4%), Ge (99.99%), B (99.4%), and Sn (99.99%) under a Zr-gettered high-purity Ar (99.999%) atmosphere. In the course of the preparation, the alloy ingot was flipped and remelted four times for homogenization. All casting trials were performed under the high-purity argon atmosphere after flushing the vacuum chamber of the melt spinner (Melt Spinner HV, Edmund Bühler) twice with argon and bringing the vacuum level down to ∼3.10^–6^ mbar. The velocity of the copper wheel was kept constant at 31.4 m/s. Ribbons were quenched from the master alloy mentioned above using quartz crucibles.

The as-produced ribbons were analyzed by X-ray diffraction to confirm their amorphous structure. These measurements were conducted in reflection configuration (D2 phaser, Bruker) using Co Kα (λ = 1.78897 Å) radiation. Differential scanning calorimetry was used to determine the glass-transition (*T*_g_) and crystallization (*T*_x_) temperatures. The tests were conducted using a Netzsch DSC 404 F1 Pegasus device under a high-purity (99.999%) Ar atmosphere at constant heating and cooling rates of 20 K/min.

The corrosion behavior of the ribbons was analyzed by electrochemical tests in a naturally aerated solution of NaCl at 0.9 wt %, with an adjusted pH = 7.4 ± 0.1 at room temperature. The electrochemical measurements were performed in a three-electrode glass cell using a reference 600 potentiostat (Gamry Instruments). A saturated calomel electrode (SCE) was used as a reference electrode and a graphite electrode as a counter electrode. Part of the ribbons was immersed in water as a working electrode. The immersed surface was delimited by a blocking varnish, and the electrical connection was isolated from the solution. The exact exposed surface was determined a posteriori by optical microscopy (∼0.1–0.5 cm^2^). The open-circuit potential was monitored for 7200 s to achieve a stationary interface between the working electrode and electrolyte. Subsequently, a cyclic polarization test was carried out with a scan rate of 1 mV/s starting from −0.2 V vs SCE. The limiting conditions for the forward scan were +2.5 V vs SCE or a current density of 2 mA/cm^2^. The reverse scan rate was 1 mV/s, and the scanning was stopped when the current density reached negative values. The tests were repeated 3 times.

### 2.2 Surface Functionalization and Characterization

Tannic acid (TA—tannic acid 403040–100 G, Sigma-Aldrich, St. Louis, MO) was dissolved in ultrapure water, and TRIS/HCl buffer was added with 292 mg/L of calcium chloride. A 5 mg/mL TA acid solution in different media was used for the functionalization. The solution was stirred for 1 h to reach the complete dissolution of the tannic acid. The TRIS/HCl-calcium chloride solution was selected to evaluate the effect of pH on the functionalization and the possibility of using Ca^2+^ ions as mediators to graft tannic acid to the surface of the metallic glass, as previously studied for the functionalization of titanium surfaces with polyphenols.^34,35^ Ti_60_Zr_20_Si_8_Ge_7_B_3_Sn_2_ ribbons were cut into 1 cm long pieces and washed in ultrapure distilled water in an ultrasonic bath: the ribbon pieces were further activated under ultraviolet (UV) irradiation for 1 h before functionalization. Each ribbon was soaked in 5 mL of TA solution (5 mg/mL) for 3 h at 37 °C in the dark. Afterward, the samples were gently washed twice in ultrapure water and dried in air in the dark.

The presence of tannic acid and its redox activity after grafting were evaluated on functionalized samples using the Folin and Ciocalteu test. This test is widely used for the quantification of total polyphenols in liquid media and was adapted to the analyses of solid samples in previous works.^36^ Briefly, each functionalized sample was soaked in 8 mL of ultrapure water and then 0.5 mL of the Folin and Ciocalteu reagent (Folin&Ciocalteu’s phenol reagent 2 M with respect to acid) and 1.5 mL of 20 w/v% Na_2_CO_3_ were added. The amount of tannic acid was quantified in gallic acid equivalents (GAE mg/mL) by UV measurements (UV-2600, Shimadzu) at 760 nm after 2 h of reaction by the calibration curve obtained with gallic acid.

The isoelectric point and the ζ-potential as a function of pH were evaluated by following the streaming potential technique using an electrokinetic analyzer (SurPASS, Anton Paar) equipped with an adjustable gap cell. The measurements were performed in 0.001 M KCl as the electrolyte, and the pH was varied by the addition of 0.05 M HCl or 0.05 M NaOH through the instrument’s automatic titration unit. The acid and alkaline sides of the curve were obtained in two different steps on the same set of samples, first testing the basic one and washing the samples between the two steps.

For comparison, ζ-potential titration curves of tannic acid solutions (diluted in water or TRIS/HCl + Ca) were obtained by using a dynamic light scattering device (DLS, Litesizer, Anton Paar) equipped with an omega cuvette for ζ-potential measurements.

The presence and distribution of tannic acid on the ribbon surface were investigated using fluorescence microscopy, exploiting the tannic acid autofluorescence.^37,38^ The autofluorescence phenomenon is based on the deactivation, from an excited electronic state to the ground state, with the spontaneous emission of light, and it is typical, for example, of aromatic compounds as phenols.^39^ A confocal microscope (LSM 900, ZEISS) equipped with a fluorescent light source (excitation wavelength of 573 nm) was used for this purpose.

X-ray photoelectron spectroscopy was performed using a Thermo Scientific Nexsa G2 Surface Analysis System (Thermo Fisher Scientific, U.K.) equipped with a microfocused, monochromatic Al Kα X-ray source (1486.68 eV). The spectra for the survey were acquired in a constant analyzer energy mode with a pass energy of 200 eV. Narrow spectral regions were collected by using a pass energy of 50 eV. Charge compensation was achieved with an Ar flood gun system. Thermo Scientific Avantage software, version 6.6.1 (Thermo Fisher Scientific), was used for digital acquisition and data processing. The surface compositions (in atomic %) were determined by considering the integrated peak areas of the detected atoms and the respective sensitivity factor.

### 2.3 Cytocompatibility Evaluation

#### 2.3.1 Cell Cultivation

The cytocompatibility analysis was conducted by primary human gingival fibroblasts (HGFs), which are regarded as the main cell type in the periimplant soft tissue. HGFs were purchased from the American Type Culture Collection (PCS-201–018 from ATCC, Manassas) and were cultivated in a minimum essential medium Eagle α modification (α-MEM, Sigma-Aldrich, Milan, Italy) supplemented with 10% fetal bovine serum (FBS, Merck, Milan, Italy) and 1% antibiotics (penicillin/streptomycin, Merck, Milan, Italy) at 37 °C, 5% CO_2_ atmosphere. Cells were cultivated until an 80–90% confluence, detached by a trypsin–EDTA solution (0.25% in phosphate buffer saline (PBS), Merck, Milan, Italy), harvested, and used for the experiments.

#### 2.3.2 Cytocompatibility Evaluation

Due to the aim of this study, which was the development of Ti_60_Zr_20_Si_8_Ge_7_B_3_Sn_2_ MG ribbons with functionalization of TA for use as abutments of dental implants, the evaluation of the possible toxic effect of the released ions (indirect assay) on the host cells (HGF) was critical. This assessment was analyzed according to the International Organization Standardization (ISO 10993–5:2009) protocol. Briefly, TA-functionalized and nonfunctionalized Ti_60_Zr_20_Si_8_Ge_7_B_3_Sn_2_ MG ribbons were immersed in 7 mL of α-MEM medium and agitated at 120 rpm at 37 °C for 7 days. At this time point, the supernatants were collected and were used to cultivate the HGF cells seeded at a defined concentration (1.5 × 10^4^ cells/well) into a 24-multiwell plate; cells cultivated with supernatants obtained from polystyrene were considered as control specimens. Subsequently, indirect cytocompatibility properties of the supernatant were investigated after 24 h; at that time point, the metabolic activity of the cells was evaluated using the cell viability reagent (alamar Blue, ready-to-use solution from Life Technologies, Milan, Italy) by directly adding the dye solution (0.015% in PBS) onto the cell-seeded specimens. After 4 h of incubation in the dark, the fluorescent signals (expressed as relative fluorescent units—RFUs) were detected at a fluorescence excitation wavelength of 570 nm and a fluorescence emission reading of 590 nm by a spectrophotometer (Spark, Tecan Trading AG, Basel, Switzerland). Moreover, the fluorescent Live/Dead assay was applied to visually check for viable cells (Live/Dead, Viability/Cytotoxicity Kit for Mammalian Cells, Invitrogen, Milan, Italy) with a digital EVOS FLoid microscope (Life Technologies, Milan, Italy). Finally, field emission electron microscopy (FE-SEM, SUPRATM 40, Zeiss) imaging was used to detect the morphology of attached and spread cells. Briefly, the specimens were dehydrated by the alcohol scale (70–90–100% ethanol for 1 h each), dried with hexamethyldisilazane, mounted onto stubs with conductive carbon tape, and covered with a chromium layer.

Additionally, the cytocompatibility properties of the TA-functionalized and nonfunctionalized Ti_60_Zr_20_Si_8_Ge_7_B_3_Sn_2_ MG ribbons were evaluated in direct contact (direct assay) with seeding the HGF cells at a defined density (1.5 × 10^4^ cells/sample) directly on the samples’ surfaces. After 4 h of incubation at 37 °C to allow HGF cells to adhere to the surfaces, 500 μL of culture medium (α-MEM + 10% FBS) was added to each sample. The viability of the surface-attached cells was investigated with the alamar blue reagent and fluorescent Live/Dead assay (as mentioned above); nonfunctionalized samples were considered a control or as-produced samples.

### 2.4 Antibacterial Evaluation

#### 2.4.1 Strain Growth Condition

The antibacterial properties of the specimens were assessed against methicillin-resistant *S. aureus* (MRSA *S. aureus–* ATCC 43300), a Gram-positive bacterium that is defined as a standard strain that is resistant to many antibiotics to evaluate the antibacterial properties of materials.^40^ Moreover, in addition to oral pathogen bacterial cells, other pathogens like *S. aureus*, which transit to the mouth from the anterior nares, could create a biofilm layer on the abutments, which subsequently leads to oral cavity and sometimes implant failure.^41,42^ The bacteria were cultivated in Trypticase Soy Agar plates (TSA, Sigma-Aldrich, Milan, Italy) and incubated at 37 °C until round single colonies were formed; then, a few colonies were collected, spotted into 15 mL of Luria-Bertani broth (LB, Sigma-Aldrich, Milan, Italy), and incubated overnight at 37 °C under agitation (200 rpm). The day after, a fresh broth culture was prepared before the experiment by diluting bacteria into a fresh medium to a final concentration of 1 × 10^3^ bacteria/mL, corresponding to a spectrophotometric optical density of 0.00001 at a 600 nm wavelength.^43^

#### 2.4.2 Antibacterial Activity Evaluation

Antiadhesive and antibacterial properties of the TA-functionalized and nonfunctionalized Ti_60_Zr_20_Si_8_Ge_7_B_3_Sn_2_ MG ribbons (as-produced or control) were assayed after 2 h (early time point) and 24 h (late time point) of direct infection, respectively; the nonfunctionalized samples were considered control specimens. Briefly, the samples were immersed into 1 mL of LB containing 1 × 10^3^ bacterial cells in a 2 mL Eppendorf tube and agitated at 200 rpm at 37 °C for 2 h (early time point) to allow bacterial cells to attach, and microcolonies were formed on the surfaces of the specimens; after this time, the samples were transferred to a sterile 24-multiwell plate, and 500 μL of fresh LB broth was added into the wells and incubated in a static condition at 37 °C for 24 h (late time point) to allow bacterial biofilm to grow. At each time point (early and late time points), the adhered bacteria were detached from the specimens’ surface by sonication (5 min, 3 times) and vortex (20 s) following a setup unable to determine bacterial death due to the mechanical detachment (amplitude 27% and frequency 39 kHz).^44^ Then, the number of viable bacteria was determined by the colony-forming unit (CFU) count as previously detailed;^45^ additionally, the viability of the adhered bacteria was evaluated through their metabolic activity using the alamar blue assay (0.0015% in PBS, as explained in Section 2.3.2). Finally, the viability and morphology of the surface-attached bacteria and surface-formed biofilm were investigated using the fluorescent Live/Dead assay (BacLight, Bacterial Viability Kit for microscopy, Invitrogen, Milan, Italy) and SEM, respectively, as detailed in Section 2.3.2. Finally, three-dimensional (3D) reconstructed SEM images were taken with SmileView software (Map 8.2.9621, JEOL, Japan): briefly, after taking a SEM image and manually removing the noises from the background by setting the minimum and maximum values, the 3D image is automatically reconstructed by the SmileView software, providing information about the thickness or height of the peaks corresponding to the bacterial colonies.

### 2.5 Statistical Analysis

All experiments were performed in triplicate. The results were statistically analyzed using the SPSS software (v.20.0, IBM). First, the normal distribution of the data and the homogeneity of the variance were confirmed by Shapiro–Wilk’s and Levene’s tests, respectively; then, the different groups were compared by the one-way ANOVA using Tukey’s test as post hoc analysis. Significant differences were established at *p* values <0.05.

## 3 Results and Discussion

### 3.1 Thermal and Structural Analyses

**Figure 1:**
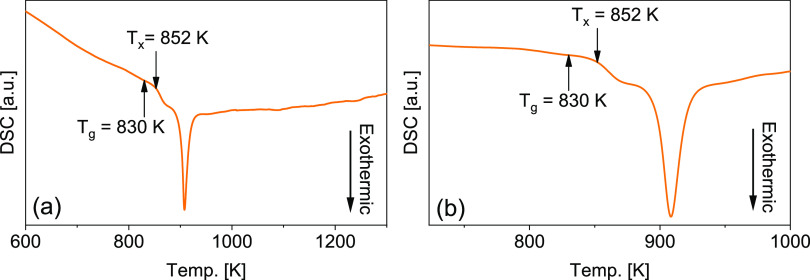
(a) High-temperature DSC scan of a melt-spun glassy ribbon registered at a heating rate of 20 K/min. (b) DSC scan was normalized via subtracting the second heating curve from the first one.

Figure 1a displays a characteristic continuous-heating DSC scan of the as-produced amorphous ribbon. The amorphous atomic structure of the glassy ribbon gives rise to a two-stage crystallization event, which is apparent from two superimposed exothermic peaks. It is also clear that the alloy crystallizes in a large temperature interval rather than in a narrow single exothermic peak, which signifies that the alloy composition is far from being eutectic. Determining the glass-transition temperature (*T*_g_) can often be problematic for melt-spun metallic glass ribbons, especially for marginal glass-forming alloys.^24^ For the fully biocompatible Ti-based amorphous alloys, many research works have reported the lack of a clear endothermic bump that could account for *T*_g_,^1,6,17,46^ which is also the case for the alloy in this study. Since the continuous DSC scan does not display a clear glass-transition event, the second heating curve was subtracted from the initial one to better visualize *T*_g_. Figure 1a,b indicates the measured *T*_g_ and *T*_x_ values of the studied alloy. It can be observed that even after the curve subtraction, the alloy possesses a relatively small endothermic bump just before crystallization. The onset (830 K) of this minor endothermic bump should account for the glass-transition temperature. A thermal GFA parameter, the extension of the supercooled liquid region (SCLR = Δ*T*_x_ = *T*_x_ – *T*_g_) of the glassy alloy, is calculated, and the result is shown in Table 1.

**Table 1:** Thermal Properties (Δ*T*_x_ = *T*_x_ – *T*_g_, *T*_g_ = Glass-Transition Temperature,*T*_x_ = Crystallization Onset Temperature) and Melt-Spinning Ejection Temperatures of the Investigated Alloys^a^

alloy	*T*_g_ [K]	*T*_x_ [K]	Δ*T*_x_ [K]	*T*_eject_ [K]
Ti_60_Zr_20_Si_8_Ge_7_B_3_Sn_2_	830	852	22	1823

a The error limit of the DSC is ±2 K.

Table 1 shows that the Ti_60_Zr_20_Si_8_Ge_7_B_3_Sn_2_ alloys exhibit a narrow SCLR of 22 K, which seems reasonable for this level of GFA. Another crucial thermal parameter for GFA, the reduced glass-transition temperature (*T*_rg_ = *T*_g_/*T*_liq_) of the alloys, cannot be calculated due to the very noisy melting signals of the alloy.

**Figure 2:**
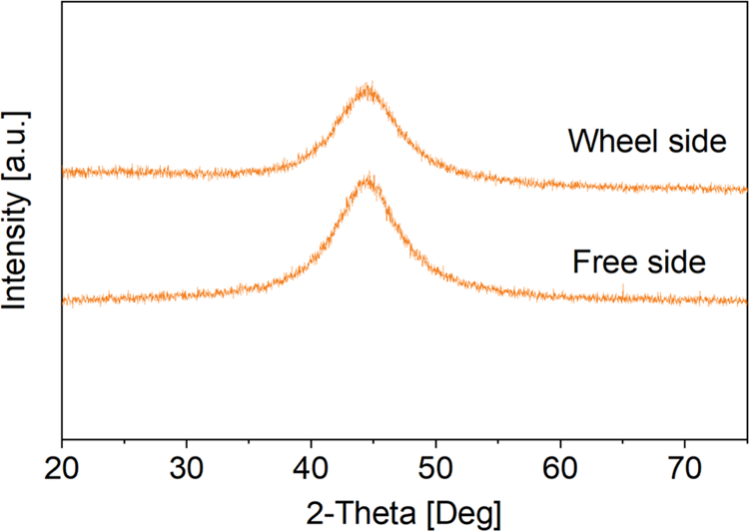
XRD patterns registered from the wheel and free sides of the as-produced ribbon.

The XRD patterns show broad diffuse diffraction maxima for the wheel- and free-side of the as-produced ribbon. The measurements were repeated by the samples taken from many different locations of the as-produced material, which confirmed the glassy structure of the alloy (Figure 2).

### 3.2 Surface Characterization

The Folin and Ciocalteu test was used for the quantification of tannic acid grafted on the ribbon surface and of its redox activity. The TA concentration in gallic acid equivalents (GAE) is zero for the bare substrates, 0.0001 ± 0.00009 mg/mL for ribbons functionalized in the water-TA solution, and 0.0258 ± 0.0035 mg/mL for ribbons functionalized in the TRIS/HCl + Ca solution. A significant improvement in the functionalization ability can be detected using the TRIS/HCl + Ca solution, suggesting a fundamental role of neutral pH and Ca^2+^ ions in TA grafting to the ribbon surface. Moreover, the positive response of the grafted TA to the F&C test evidences the maintenance of its redox activity after grafting.

**Figure 3:**
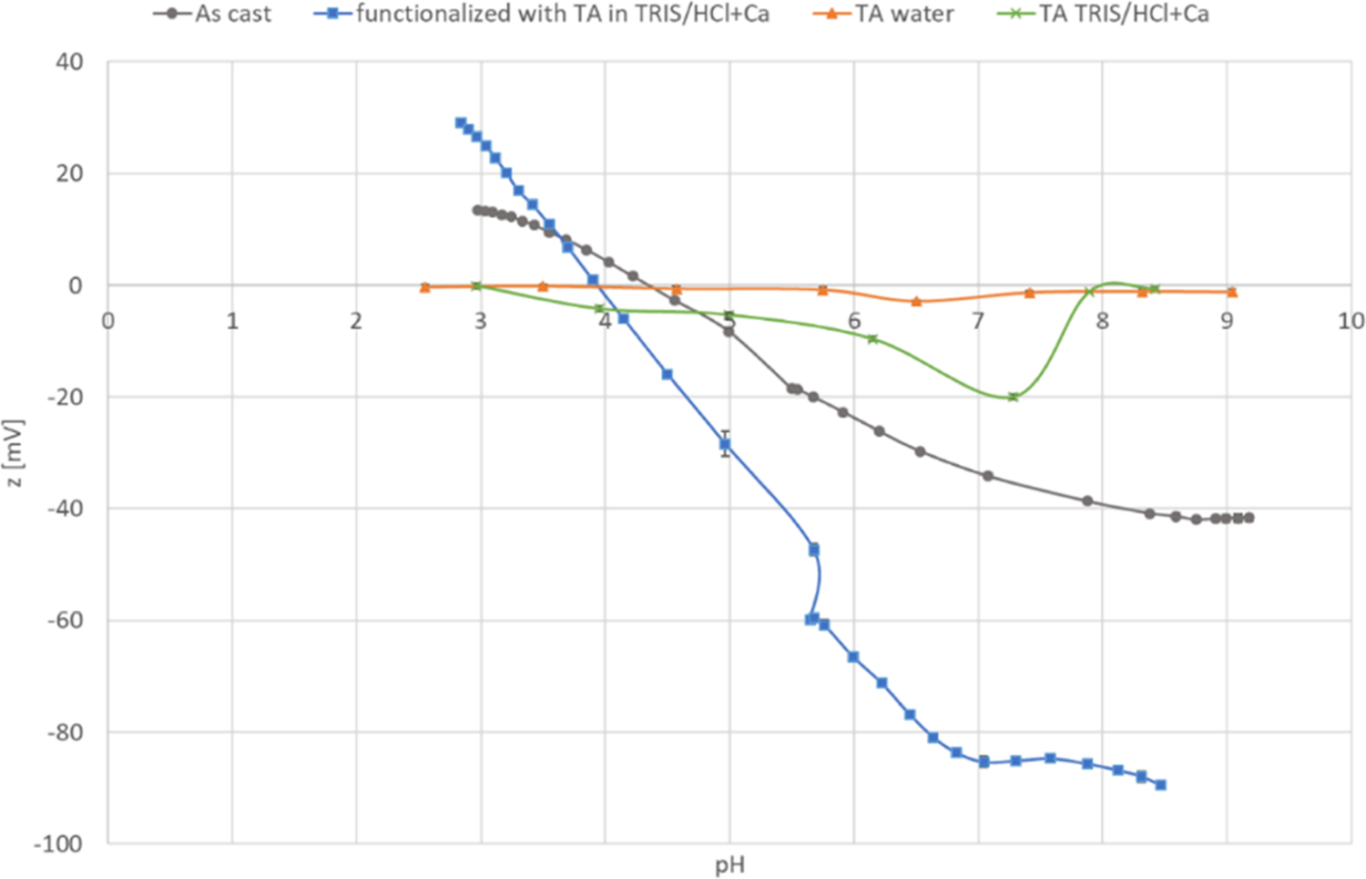
ζ-potential measurements on as-produced and TA-functionalized Ti_60_Zr_20_Si_8_Ge_7_B_3_Sn_2_ MG ribbons.

To confirm the presence of TA grafted on the surface of the Ti_60_Zr_20_Si_8_Ge_7_B_3_Sn_2_ ribbons, samples were also investigated by ζ-potential measurements. The titration curves of the bare substrate, samples functionalized in the TRIS/HCl + Ca TA solution, and TA solutions prepared in water and TRIS/HCl + Ca are shown in Figure 3. The standard deviation of the measurements is very small, suggesting surface stability in the explored pH range. The isoelectric point (IEP) of the as-produced ribbon is 4.34, not far from the one reported in the literature for titanium and its alloys.^47^ This IEP value is typical of surfaces without the significant presence of acidic or basic functional groups. After functionalization, the IEP value shifts to more acidic values (3.93), indicating that a surface modification occurred, which is in accordance with the acidic IEP of TA. In fact, the IEP of TA, both in TRIS/HCl + Ca and in water, is close to 3, a value analogous to the one reported in the literature for the same molecule.^48^ The presence of acidic functional groups (the OH groups of the TA molecule) on the functionalized surface is confirmed by the appearance of a plateau in the basic region (starting from pH 7). A plateau in a ζ-potential titration curve occurs when a specific type of functional group with a single *K*_a_ value is exposed to the solution. This plateau is almost absent on the bare substrate (only a small plateau from pH 9 can be noticed on the as-cast ribbon curve, attributable to a small amount of acidic OH groups on the surface with low acidic strength). Looking at the ζ-potential curves of the substrate and the solutions, it can be evidenced that the TA molecules in the solution have a ζ-potential close to 0 and the ribbon surface is weakly positive (ζ ≅ 10 mV) when the functionalization is performed in water (pH of the TA solution in water is 3.50), suggesting a negligible electrostatic interaction between the TA molecules and the substrate. On the other hand, the tannic acid solution is slightly negative (ζ ≅ −20 mV) and the ribbon surface is much more negative (ζ ≅ −35 mV) when the functionalization is performed in the TRIS/HCl + Ca solution (pH close to 7.4). It can be supposed that, in these conditions, the ribbon surface is prone to the adsorption of Ca^2+^ ions and subsequent grafting of TA molecules, with Ca^2+^ ions acting as a linker, as previously observed for polyphenols grafting on titanium surfaces.^35^

Since tannic acid, as many polyphenols, is autofluorescent,^37,38^ the as-produced and functionalized ribbons were visualized by a confocal microscope equipped with a fluorescent source; the results are shown in Figure S1. The fluorescence signal, attributable to the TA molecules, is detectable only on functionalized ribbons. The here-reported surface modification is a functionalization that produces the grafting of a thin (molecular) layer of TA without any modification of the surface roughness and morphology, as previously observed by the authors on different titanium substrates.^31,32^

### 3.3 Compositional and Chemical State Analysis

To understand the tannic acid effect in metallic glass samples, XPS was carried out. The XPS scans were recorded for as-produced 0 s, as-produced 300 s argon-etched samples, TA-functionalized 0 s, and TA-functionalized 300 s argon-etched Ti_60_Zr_20_Si_8_Ge_7_B_3_Sn_2_ samples. The XPS survey spectra of the considered samples are shown in Figure S2. The peaks of Ti2p, Zr3d, C1s, and O1s were observed in the XPS survey spectrum.

**Figure 4:**
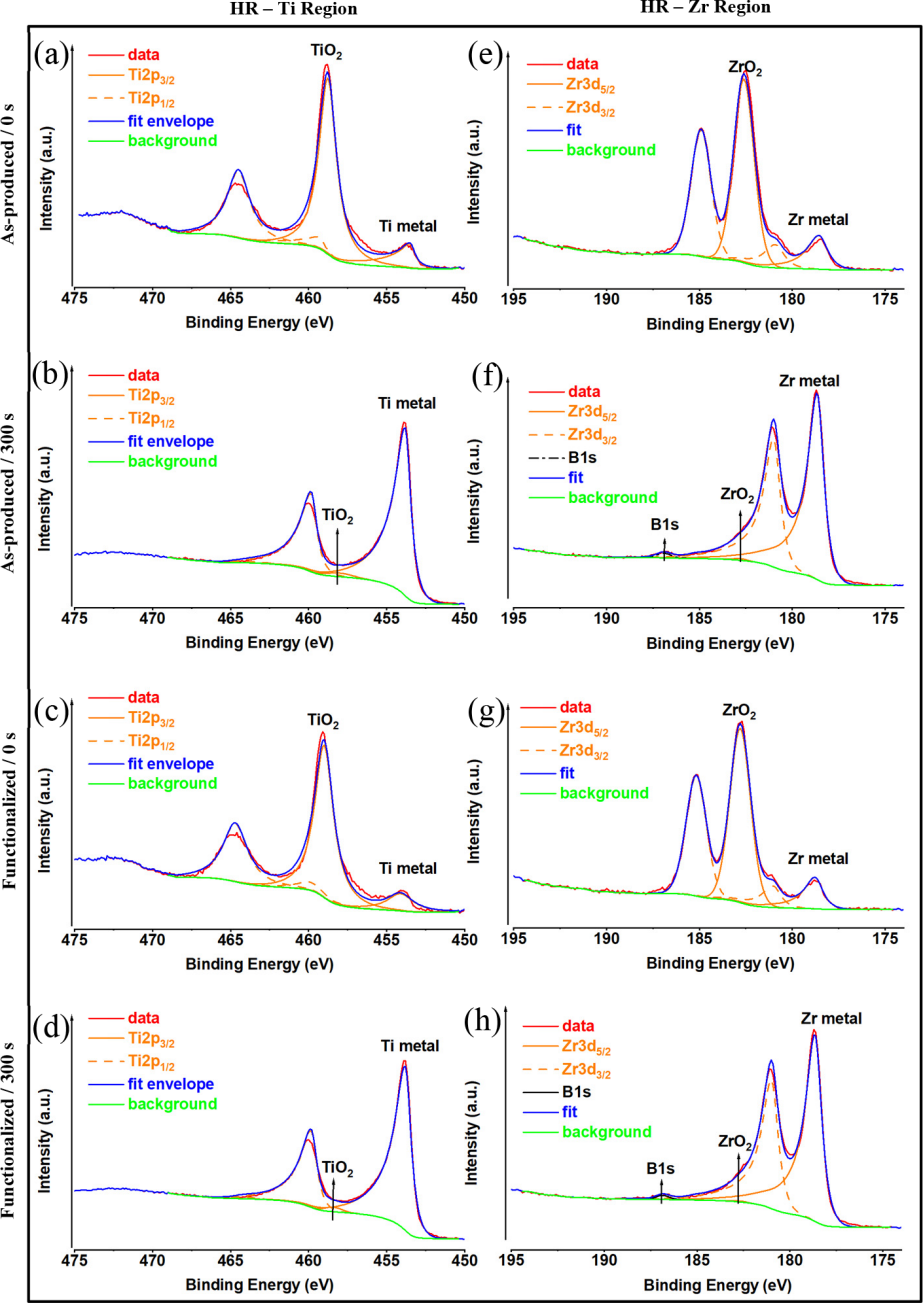
High-resolution Ti and Zr XPS analysis of Ti_60_Zr_20_Si_8_Ge_7_B_3_Sn_2_ MG ribbon surfaces for as-produced (a, e), as-produced and 300 s argon-etched (b, f), TA-functionalized (c, g), and TA-functionalized and 300 s argon-etched (d, h) samples.

**Figure 5:**
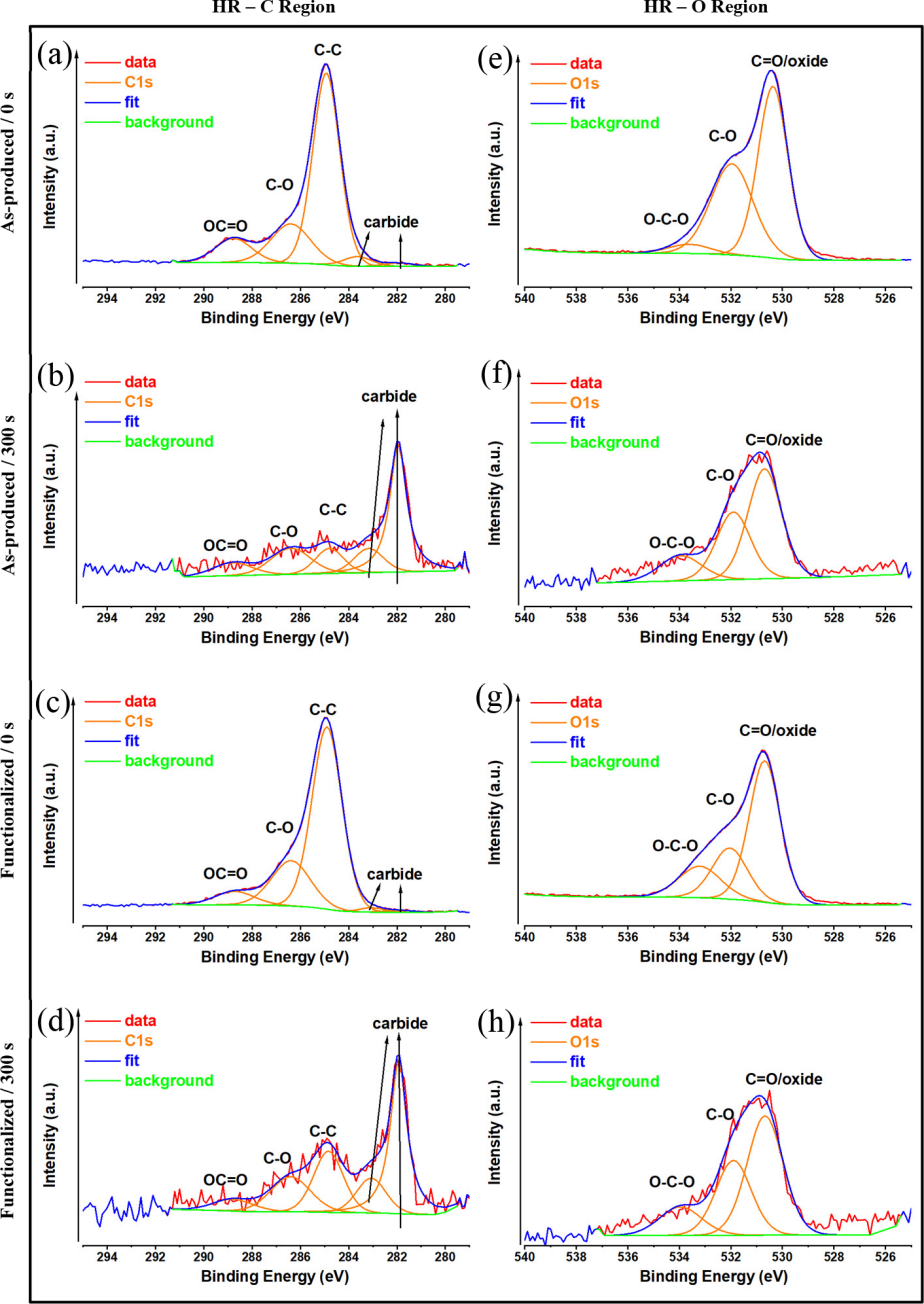
High-resolution C and O XPS analysis of Ti_60_Zr_20_Si_8_Ge_7_B_3_Sn_2_ MG ribbon surfaces for as-produced (a, e), as-produced and 300 s argon-etched (b, f), TA-functionalized (c, g), and TA-functionalized and 300 s argon-etched (d, h) samples.

Figure 4a–d displays the curve fittings of the high-resolution XPS spectra of Ti2p^3^ core levels for all samples. The peaks at 453.5 and 458.8 eV are attributed to the Ti2p^3^ metal^49^ and Ti2p^3^ TiO_2_,^50^ respectively. The XPS spectra of all of the samples at 178.6^51^ and 186.2 eV are related to the Zr3d^5^ metal and Zr3d^5^ ZrO_2_^52^ structures, respectively (Figure 4e–h). The compositional analysis of all samples is provided in Table S1. The MG surface contains carbon, which is due to air and hydrocarbon impurities that are usually present on titanium surfaces,^53^ but it is mostly composed of Ti and O (Table S1). Most of the carbon is due to surface pollution, and after 300 s of etching, it nearly completely vanishes (Figure 5b,d).

As expected, tannic acid consists mainly of C and O. For this reason, the amount of C and O increases on the surface functionalized with tannic acid (Table S1). Additionally, the XPS scan of TA-functionalized MG samples (0 and 300 s) displays a C 1s peak at ∼284 eV (Figure 5c,d) and an O 1s peak at ∼530 eV (Figure 5g,h), respectively. The amount of the C-O/OCO species at 286.4 and 288.5 eV probably defines the ester groups and ether groups in tannic acid.^54,55^ The loss of oxygen-containing functional groups, like –COOH and –OH, points to the hydrolysis of tannic acid and may be inferred as tannic acid is the primary source of O element in the oxide layer.^56^

### 3.4 Electrochemical Properties

**Figure 6:**
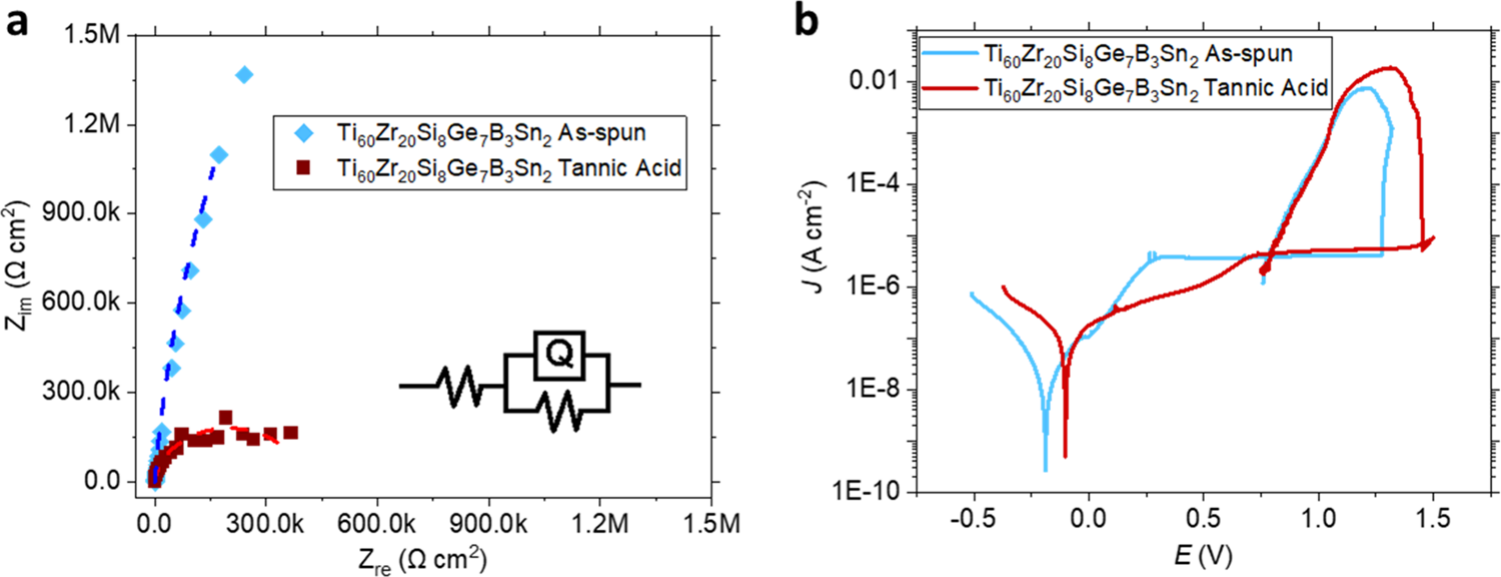
(a) Nyquist plots of as-produced and tannic acid-functionalized Ti_60_Zr_20_Si_8_Ge_7_B_3_Sn_2_ ribbons. The data are fitted by the *R*(*QR*) circuit (inset) indicated by the corresponding dashed lines. (b) Potentiodynamic polarization results of the corresponding samples.

The corrosion resistance of the Ti_60_Zr_20_Si_8_Ge_7_B_3_Sn_2_ ribbons was investigated by electrochemical impedance spectroscopy and cyclic potentiodynamic polarization tests. The influence of the double-layer capacitance (Figure 6a), i.e., *Q*–*Y*_o_ and *Q*–*n*, is small. This is explained by the oxide layer on the as-produced state sample as well as the tannic acid coating preventing ionic build-up on the working electrode surface. On the other hand, the charge-transfer resistance, *R*_ct_, decreases due to the interaction of tannic acid with the Ti-rich oxide layer (Table 2). Tannic acid can be used as a surface modifier that enhances stability and improves corrosion properties. This is corroborated by Figure 6b, where the point reaching the passivation, *E*_pass_, and the corrosion potential, *E*_corr_, shift toward positive values after tannic acid treatment. Furthermore, the passivation regions η_pit_ and *E*_pit_–*E*_rp_ increase after the tannic acid treatment (Table 3). The corrosion current density of the measured samples is several orders of magnitude lower as compared to Ti–6Al–4V in untreated and thermally oxidized states, and the passivation current is also an order of magnitude lower as compared to the untreated state,^57^ corroborating its suitability for the corrosion tests.

**Table 2:** EIS Data Fitted by the *R*(*QR*) Equivalent Circuit Model

results	*E*_corr_	*J*_corr_	*J*_pass_	*E*_pit_	*E*_rp_	η_pit_	*E*_pit_–*E*_rp_
unit	[V vs Ag/AgCl]	10^–9^ [A/cm^2^]	10^–6^ [A/cm^2^]	[V vs Ag/AgCl]	[V vs Ag/AgCl]	[V vs Ag/AgCl]	[V vs Ag/AgCl]
as-produced	–0.188 ± 0.001	7.758 ± 0.057	3.918 ± 0.005	1.277 ± 0.001	0.787 ± 0.010	1.465 ± 0.010	0.490 ± 0.010
TA-functionalized	–0.101 ± 0.001	35.025 ± 0.038	4.393 ± 0.020	1.451 ± 0.001	0.795 ± 0.004	1.552 ± 0.001	0.656 ± 0.004

**Table 3:** Electrochemical Results for the Ti_60_Zr_20_Si_8_Ge_7_B_3_Sn_2_ MG Ribbons with and without Tannic Acid^a^

*R* (*QR*)	as-produced	TA-functionalized
*R*_s_ (Ω·cm^2^)	13.02 (%3.85)	11.84 (%1.62)
*Q–Y*_o_ (Ss*^n^* cm^–2^)	1.327 × 10^–5^ (%3.02)	1.384 × 10^–5^ (%1.45)
*Q–n* (−)	0.961 (%0.65)	0.940 (%0.30)
*R* (Ω·cm^2^)	1.230 × 10^7^ (%116.9)	4.000 × 10^5^ (%3.22)
χ^2^	2.762 × 10^–2^	4.500 × 10^–3^

a *E*_corr_ = corrosion potential, *J*_pass_ = passivation current density, *E*_pit_ = pitting potential, *E*_rp_ = repassivation potential, η_pit_ = *E*_pit_ – *E*_corr_ = passivation region.

### 3.5 Cytocompatibility Properties

#### 3.5.1 Indirect Cytocompatibility Evaluation

Transmucosal parts of the dental implants (abutments) are part of the implant body in direct connection with the bone screw and the crown that is finally mounted on it; so, unlike the implant body, the connection between the abutment and soft tissue (gingiva) is minimized even if tight sealing between them is requested to prevent bacterial infection and the loss of the implant due to mechanical stress. So, the release of toxic compounds from the abutment to the surrounding gingiva must be prevented to allow for a tight adhesion. Corrosion is one of the most common reasons for materials’ degradation^58^ since the mouth environment is physiologically maintained at a pH of ∼5–6.

**Figure 7:**
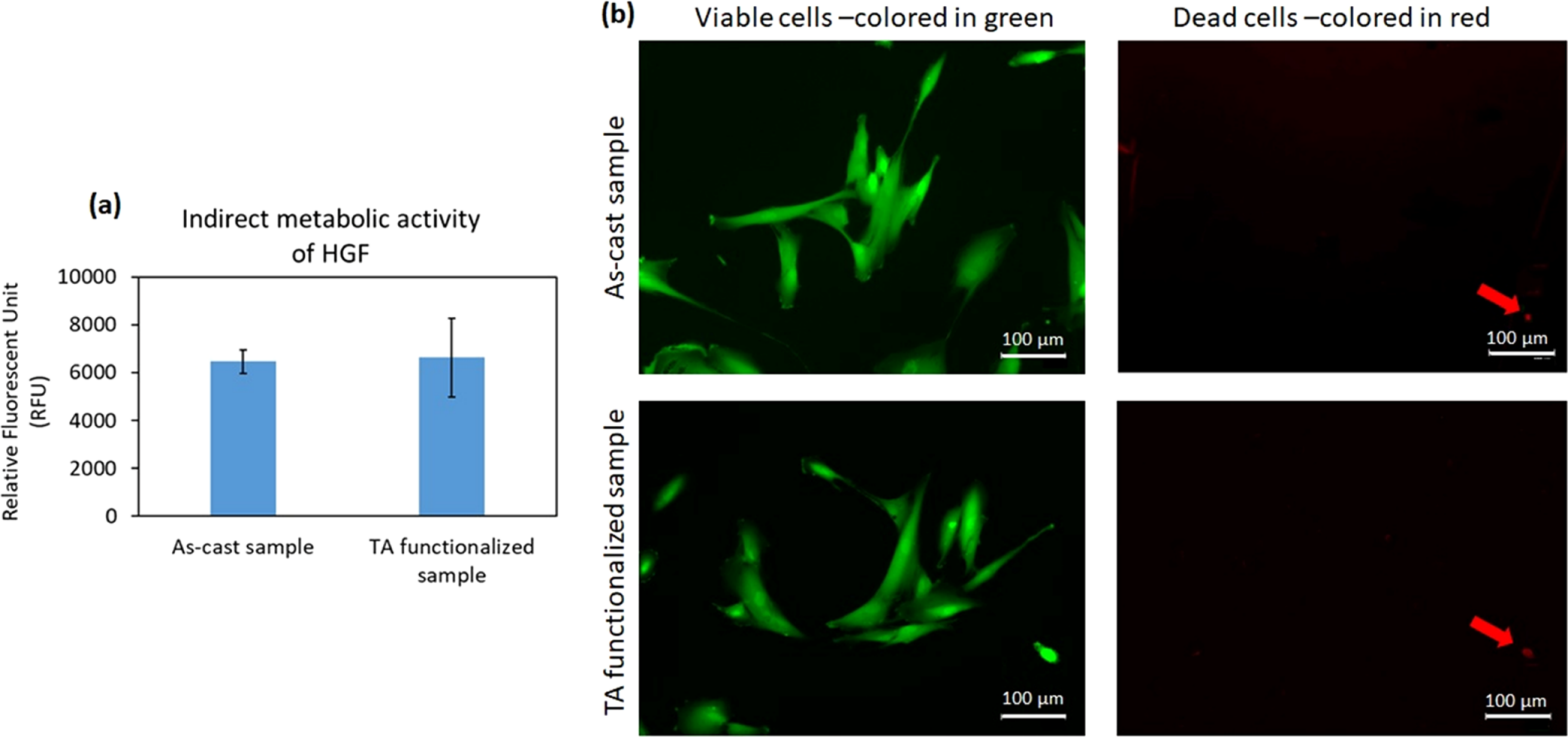
Indirect cytocompatibility evaluation of Ti_60_Zr_20_Si_8_Ge_7_B_3_Sn_2_ MG ribbon surfaces on HGF cells after 24 h. (a) Metabolic activity of cells and (b) live/dead fluorescent stain; the **left panel** demonstrates the viable cells that were colored in green, and the **right panel** shows the dead cells with red color. Arrows indicate the dead cells; scale bar = 100 μm.

To investigate whether the hypothesis that tannic acid can help in preventing surface corrosion and the relative release of toxic compounds is true, an indirect cytocompatibility evaluation of the samples was performed on primary human gingival fibroblasts following the ISO 10993–5; 2009 protocol. Accordingly, the TA-functionalized and as-produced specimens were submerged in 7 mL of α-MEM for 7 days, and their supernatants were used to cultivate HGF cells in a defined number into a 24-multiwell plate. After 24 h of incubation, the metabolic activity and the morphology of cells were analyzed by alamar blue assay and fluorescent Live/Dead staining, respectively. The results are presented in Figure 7, indicating that no statistically significant differences in terms of metabolic activity of HGF were observed between TA-functionalized Ti_60_Zr_20_Si_8_Ge_7_B_3_Sn_2_ MG ribbons and nonfunctionalized ones as control samples (as-produced sample; Figure 7a; *p* > 0.05). As the metabolic activity of the HGF cells cultivated onto polystyrene was statistically similar (*p* > 0.05) to the cells in the presence of as-cast samples, these nonfunctionalized ribbons were considered as control specimens (Figure S3). These results were confirmed visually by the fluorescent Live/Dead assays, which demonstrated that the cells cultivated with preconditioned supernatants were mostly viable (>95%, stained in green), and they proliferated properly inside the wells of the multiwell plate (Figure 7b). These findings confirmed that the chemicals used to functionalize the Ti_60_Zr_20_Si_8_Ge_7_B_3_Sn_2_ MG ribbons were not toxic for cells as well as intrinsically anticorrosion, as previously reported in the OCP and cyclic potentiodynamic polarization assays. In fact, for the functionalization of the MG ribbon, Ca^2+^ was utilized as a mediator between the surface of the samples and the layer of tannic acid; tannic acid contains a large number of pyrogallol groups that can bind strongly with the positively charged ions (Ca^2+^ and Mg^2+^) and form chelate connections to improve the corrosion resistance potential of the functionalized samples.^59,60^ So, the results from the indirect assay suggested that in the required period of 7 days expected by the ISO standard, no toxic elements coming from such surface functionalization were detected, hindering cells’ viability. This represents a promising result concerning the lack of release of toxic chemicals from the abutment to the sealing gingiva in view of the potential application of surface treatment for dental abutments.

After verification of the safety of the TA-functionalized Ti_60_Zr_20_Si_8_Ge_7_B_3_Sn_2_ MG ribbons and as-produced specimens in indirect assessment, the cytocompatibility of the samples seeded directly with cells was evaluated to simulate the adhesion and sealing of the gingival fibroblasts onto the abutment surface. Accordingly, HGF cells were directly cultivated onto the samples’ surfaces, and after 24 and 48 h of incubation, metabolic activity and viability of the surface-attached cells were performed using alamar blue and fluorescent Live/Dead assays. The results are presented in Figure S4a,b. According to metabolic activity results, after 24 and 48 h, no statistically significant differences were observed between the adhered cells onto the surfaces of the TA-functionalized Ti_60_Zr_20_Si_8_Ge_7_B_3_Sn_2_ MG ribbons in comparison with as-produced samples (*p* > 0.05, Figure S4a). Moreover, visual analysis of the attached HGF cells onto the specimens’ surfaces with fluorescent Live/Dead stain revealed that the majority (>95%) of the HGF cells were stained in green, and a low amount of red-stained dead cells were observed, indicating that the majority of surface-attached cells were viable (Figure S4b). Morphology and proliferation of the cells onto the samples’ surfaces, as-cast and TA-functionalized ribbons, were confirmed by SEM images taken after 48 h of incubation at 37 °C (Figure S4c). These results show that TA-functionalized Ti_60_Zr_20_Si_8_Ge_7_B_3_Sn_2_ MG ribbons are safe as transmucosal parts of dental implants (abutments) for adhesion of the HGF cells and especially for the formation of a tight sealing with the abutment that is fundamental to preserving the implant site from infection.^61^ To explain the cell-friendly behavior of the functionalized specimens, according to the previous studies, pyrogallol groups exposed by the tannic acid can enhance protein adsorption in the early adhesion phase as well as in the subsequent deposition of extracellular matrix (ECM); so, as a direct result, we hypothesize that cells are promoted to attach to the abutment surface through hydrogen bonds, electrostatic, and hydrophobic interactions.^62^ Therefore, the functionalization presented here did not report any toxic effect preventing cells’ adhesion and spread. On the contrary, the sealing of the soft gingival tissue can be even ameliorated by the presence of the above-mentioned chemical bonds, providing a better sealing with the abutment.

### 3.6 Antibacterial Activity Evaluation

**Figure 8:**
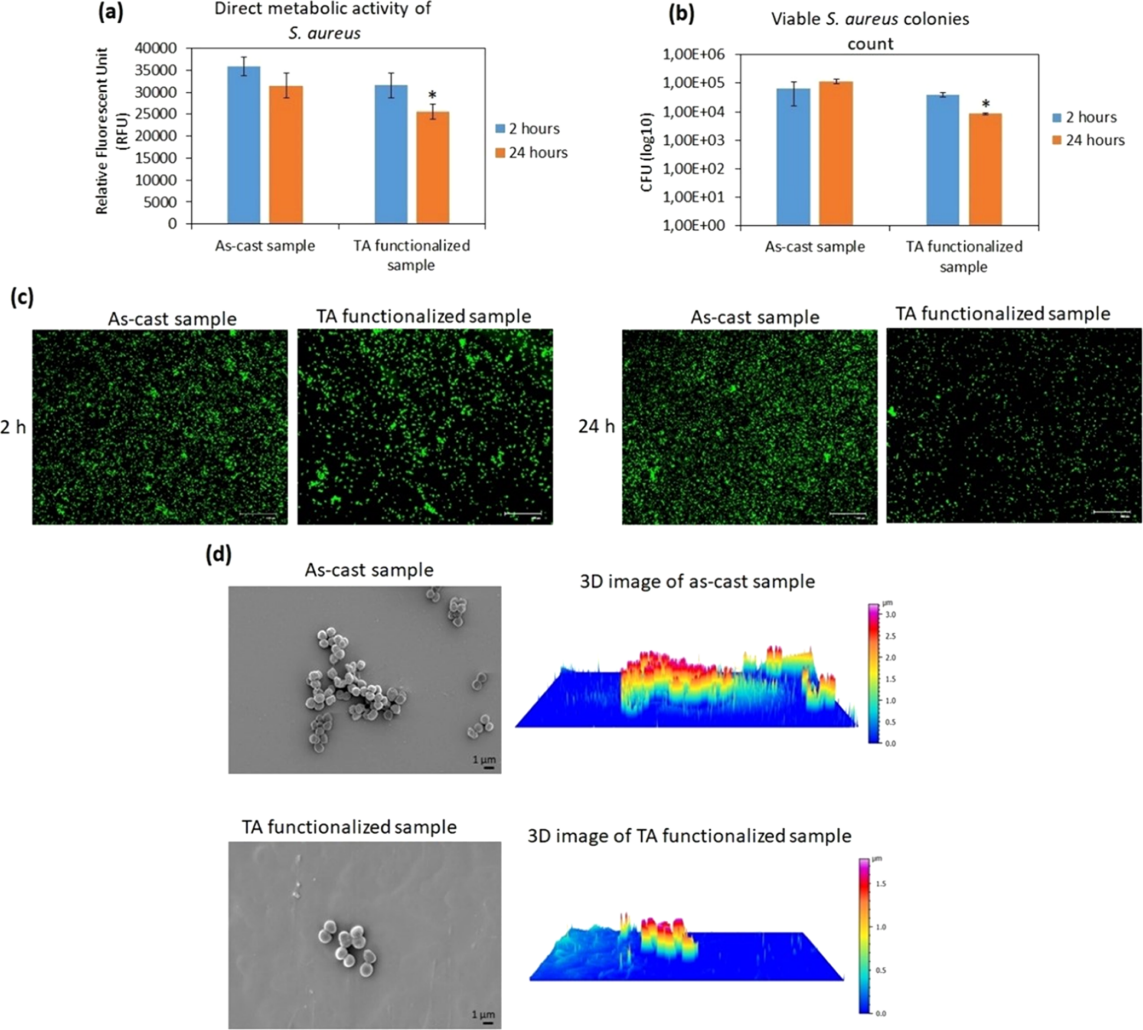
Direct antiadhesive and antibacterial evaluation of TA-functionalized Ti_60_Zr_20_Si_8_Ge_7_B_3_Sn_2_ MG ribbons’ surfaces toward *S. aureus* after 2 and 24 h. (a) Metabolic activity of surface-adherent bacterial cells; (b) viable surface-adherent bacterial colony count (CFU); (c) live/dead fluorescent stain; scale bar is 100 μm; and (d) left panel: FE-SEM images after 24 h; scale bar is 1 μm; right panel: 3D-reconstructed SEM image made with SmileView software (Map 8.2.9621, JEOL, Japan). * indicates *p* < 0.05.

In this study, methicillin-resistant *S. aureus* (MRSA) was selected as a standard multidrug-resistant strain for evaluation of the potency of the antibacterial activity of materials and antibiotics. Moreover, it can transfer from the anterior nares to the mouth and create a biofilm on the surface of abutments; as a result, it can be one of the main causes of chronic and treatment-refractory infections in many patients with dental implants.^9^ To investigate the antiadhesive and antibacterial activities of the TA-functionalized Ti_60_Zr_20_Si_8_Ge_7_B_3_Sn_2_ MG ribbons, the surface of the samples was infected directly with *S. aureus* and incubated at 37 °C for 2 h (early time point for antiadhesive evaluation) and 24 h (late time point for antibacterial evaluation). The results of the metabolic activity, counting viable bacterial colonies, and visual analysis of the bacterial cells (fluorescent Live/Dead stain and SEM) are presented in Figure 8a–d. After 2 h, no statistically significant difference in the metabolic activity of adherent bacterial cells on TA-functionalized Ti_60_Zr_20_Si_8_Ge_7_B_3_Sn_2_ MG ribbons’ surfaces was observed in comparison to that on nonfunctionalized Ti_60_Zr_20_Si_8_Ge_7_B_3_Sn_2_ MG ribbons (as-produced sample). After 24 h of incubation, the metabolic activity of bacterial cells on TA-functionalized samples was reduced to 70% in comparison with surface-attached bacterial cells on the as-produced (control) samples (*p* < 0.05 indicated by *; Figure 8a). The viable *S. aureus* colony count (CFU) revealed that after 2 h, the number of bacterial colonies that attached to the TA-functionalized Ti_60_Zr_20_Si_8_Ge_7_B_3_Sn_2_ MG ribbons’ surfaces was comparable to the number of colonies observed on the nonfunctionalized samples. However, similar to bacterial metabolic activity evaluation, after 24 h of incubation, the number of surface-adherent *S. aureus* colonies on the TA-functionalized specimens decreased approximately 1 log less than the attached bacterial colonies on the control samples’ surfaces (*p* < 0.05 indicated by *; Figure 8b). These results were visually confirmed by fluorescent Live/Dead stain (Figure 8c) and FE-SEM images (Figure 8d). As shown in Figure 8c,d, in line with the results obtained from metabolic activity and CFU, the number of viable cells attached to the surface of TA-functionalized Ti_60_Zr_20_Si_8_Ge_7_B_3_Sn_2_ MG ribbons was comparable to the number of bacterial cells on the as-produced (control) samples. However, after 24 h, Ti_60_Zr_20_Si_8_Ge_7_B_3_Sn_2_ MG ribbons functionalized with tannic acid decreased significantly (*p* < 0.05) viable attached bacterial cells on their surfaces. As shown in Figure 8d, left panel, some bacterial microcolonies (biofilm-like aggregations) were observed on the surface of as-produced samples; 3D-reconstructed SEM images, which were taken using SmileView software (MAP 8.2.9621, JEOL, Japan), showed that the height of these microcolonies was about 3.5 μm (Figure 8d, right panel). Based on the dimension of *S. aureus*, approximately 0.5–1.5 μm, it means the biofilm-like structure contains 3 or 4 layers of bacterial cells aggregated in a biofilm-like structure. However, on the surface of the TA-functionalized MG ribbons, only a few single cells were detected (with 1.5 μm in height). It is a promising result, indicating that the functionalization of Ti_60_Zr_20_Si_8_Ge_7_B_3_Sn_2_ MG ribbons with tannic acid reduces the thickness of bacterial biofilm-like structures to single and random colonies; development of anticorrosion and antibacterial materials by modifying their surfaces’ properties or coating them was reported in the previous literature.^63−65^ Despite bacterial aggregations, single colonies are very sensitive to antibiotics, and they can be easily removed from the transmucosal parts of dental implants using antiseptic mouthwash containing, for example, chlorhexidine gluconate, cetylpyridinium, and essential oils.^66^ So, it can be speculated that this antiaggregation effect can be due to the presence of the tannic acid, as previously shown by Schestakow et al.;^67^ in fact, they reported a reduction of bacterial viability and surface-adhered bacterial cells on dentin specimens by rinsing them with a solution containing tannic acid due to the interaction of bioactive groups in tannic acid with bacterial cell membranes and proteins.

## 4 Conclusions

In the current study, we have successfully fabricated Ti_60_Zr_20_Si_8_Ge_7_B_3_Sn_2_ metallic glass ribbons without any toxic elements. We have confirmed their amorphous nature using XRD and DSC. The corrosion current density of the developed alloy is several orders of magnitude lower as compared to that of Ti–6Al–4V in their untreated and thermally oxidized states, and the passivation current is also an order of magnitude lower as compared to that of the untreated state. The double-layer capacitance of tannic acid-coated samples obtained from fitting electrochemical impedance spectroscopy data is relatively smaller than for the as-produced ribbon, which is mainly due to the prevention of ionic build-up on the surface after tannic acid treatment. This is an important factor for metallic implants because it prohibits the possibility of ion release into the body or compromises its mechanical properties over time.

The ribbons were further functionalized by tannic acid to improve their performance in contact with cells and possibly prevent the growth of bacterial infection on the surface of the implant. In this regard, tannic acid dissolved in the TRIS/HCl-calcium chloride solution was used to graft TA molecules on the ribbon surface. In this case, Ca^2+^ ions act as mediators to graft tannic acid to the surface of the metallic glass, similar to previous studies for the functionalization of titanium surfaces by polyphenols. The Folin and Ciocalteu test was used for the quantification of tannic acid grafted on the ribbon surface and of its redox activity. The results indicate a significant improvement in the functionalization ability using the TRIS/HCl + Ca solution, suggesting a fundamental role of neutral pH and Ca^2+^ ions in TA grafting to the ribbon surface. ζ-potential measurements revealed a shift in the isoelectric point of the as-produced ribbon from 4.34 to more acidic values (3.93), confirming the success of surface modification by the acidic IEP of TA. The presence of acidic functional groups (the OH groups of the TA molecule) on the functionalized surface was confirmed by the appearance of a plateau in the basic region (starting from pH 7). According to the XPS results, the loss of oxygen-containing functional groups like –COOH and –OH points to the hydrolysis of tannic acid, which is possibly the primary source of oxygen in the corrosion layer.

The indirect cytocompatibility of Ti_60_Zr_20_Si_8_Ge_7_B_3_Sn_2_ MG ribbons was evaluated in vitro on primary human gingival fibroblasts, indicating no ions or wear released from the samples’ surfaces to have a toxic effect on the cells for 7 days as suggested by the ISO 10993–5:2009 protocol. Moreover, according to the direct cytocompatibility assessment, the HGF cells were safe and viable in direct contact with the surfaces of the samples, and no statistically significant differences were observed between TA-functionalized Ti_60_Zr_20_Si_8_Ge_7_B_3_Sn_2_ MG ribbons and as-produced samples (*p* > 0.05). Moving toward the antibacterial behavior of the TA-functionalized MG ribbons, after 24 h, TA-functionalized samples demonstrated a statistically significant reduction of about 30% and 1 log in metabolic activity and the number of viable attached *S. aureus* bacterial cells, respectively, in comparison with as-produced (control) samples (*p* < 0.05). Visual analysis with SEM and 3D-reconstructed images showed a few single cells on the surface of TA-functionalized samples, while on the surface of the as-produced samples, bacterial microcolonies (biofilm-like structure), which were 3–4 μm in thickness and equals 3 or 4 layers of *S. aureus*, were detected.

## Supporting Information Available

Fluorescence images of a Ti_60_Zr_20_Si_8_Ge_7_B_3_Sn_2_ as-cast ribbon (a) and after functionalization with TA (b) (Figure S1); XPS survey spectra of the Ti_60_Zr_20_Si_8_Ge_7_B_3_Sn_2_ surface for the as-cast sample (a), after 300 s etched as-cast sample (b), TA-functionalization sample (c), and after 300 s etched TA-functionalization sample (d) (Figure S2); comparison of the metabolic activity of the HGF cells cultivated onto the polystyrene with attached cells to the as-cast samples’ surfaces, *p* > 0.05 (Figure S3); and direct cytocompatibility evaluation of Ti_60_Zr_20_Si_8_Ge_7_B_3_Sn_2_ MG ribbons on the HGF cells: (a) metabolic activity of the cells after 24 and 48 h; (b) fluorescent live/dead assay after 48 h; the left panel shows the viable cells, which were colored in green, and the right panel indicates the dead cells in red color. Arrows indicate the dead cells. Scale bar = 100 μm; and (c) SEM images taken after 48 h of incubation from the surface-attached cells. Scale bar = 10 μm (Figure S4) (PDF)
