# Supplementary material for: Anticorrosion and Antimicrobial Tannic Acid-Functionalized Ti-Metallic Glass Ribbons for Dental Abutment
Source: ACS Appl Bio Mater. 2024 Feb 1;7(2):936–49. doi: 10.1021/acsabm.3c00948 (PMC10880059; doi:10.1021/acsabm.3c00948)
Supplement: Supplementary file 1 — mt3c00948_si_001.pdf [file mt3c00948_si_001.pdf]

Supplementary materials

Anti-corrosion and antimicrobial Tannic Acid functionalized Ti-metallic glass ribbons for dental abutment

Eray Yüce[1,2], Elham Sharifikolouei[3*], Matej Micusik[4], Sara Ferraris[3], Reza Rashidi[5], Ziba Najmi[5], Selin Gümrükçü[6], Alessandro Scalia[5], Andrea Cochis[5], Lia Rimondini[5], Silvia Spriano[3], Maria Omastova[4], Abdulkadir Sezai Sarac[7], Jürgen Eckert[1,2], Baran Sarac[1]**

Supplementary Figure 1: Fluorescence images of a $Ti_{60}Zr_{20}Si_8Ge_7B_3Sn_2$ as cast ribbon (a) and after functionalization with TA (b).

(a) **(b)**

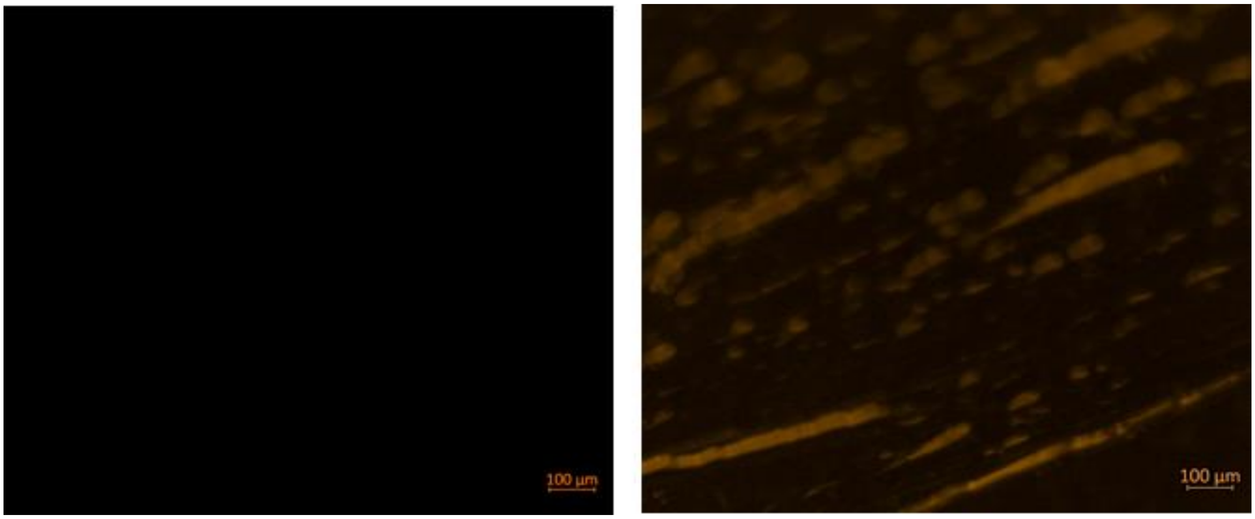

Figure S1. Fluorescence images of a $Ti_{60}Zr_{20}Si_8Ge_7B_3Sn_2$ as cast ribbon **(a)** and after functionalization with TA **(b)**.

Supplementary Figure 2: XPS survey spectra of $Ti_{60}Zr_{20}Si_8Ge_7B_3Sn_2$ surface for the as-cast sample (a), after 300 seconds etched as-cast sample (b), TA-functionalization sample (c), and after 300 seconds etched TA-functionalization sample (d).

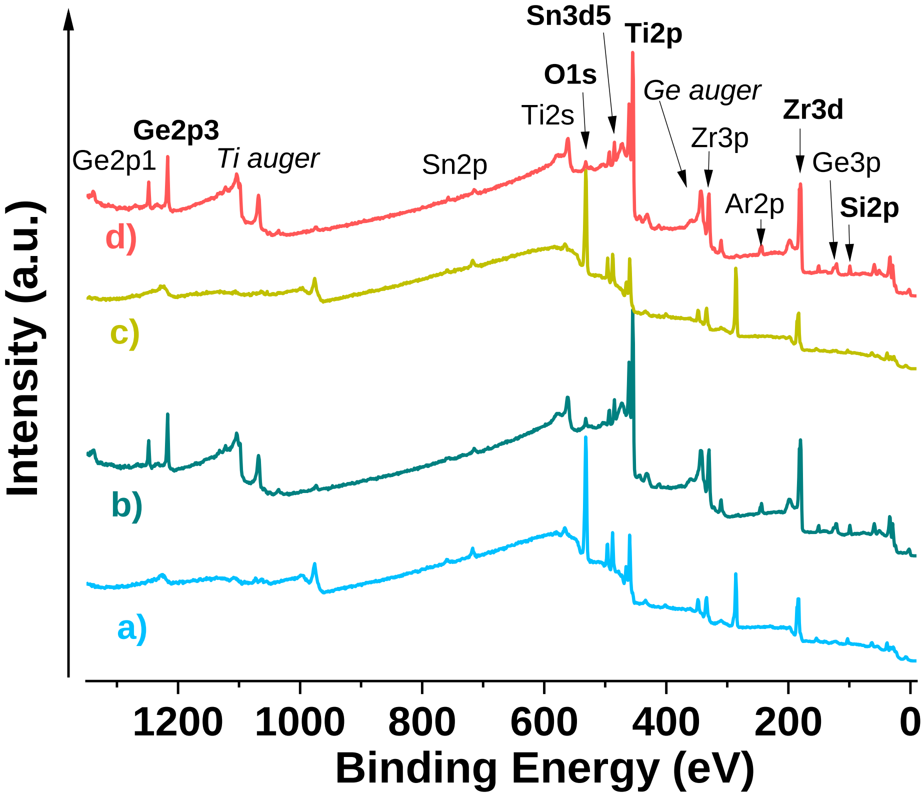

Figure S2. XPS survey spectra of $Ti_{60}Zr_{20}Si_8Ge_7B_3Sn_2$ surface for the as-cast sample **(a)**, after 300 seconds etched as-cast sample **(b)**, TA-functionalization sample **(c)**, and after 300 seconds etched TA-functionalization sample **(d)**.

Supplementary Figure 3: Comparison of the metabolic activity of the HGF cells cultivated onto the polystyrene with as-cast samples

Prior to evaluating the cytocompatibility properties of the non-functionalized $Ti_{60}Zr_{20}Si_8Ge_7B_3Sn_2$ MG and TA-functionalized $Ti_{60}Zr_{20}Si_8Ge_7B_3Sn_2$ MG ribbons towards primary Human Gingival Fibroblast cells (HGF), the metabolic activity of HGF cells cultivated onto the polystyrene considered, as gold standard, was compared to the cells attached to the non-functionalized samples (as-cast) by measuring the fluorescent intensity of the metabolized alamar blue reagent at an emission wavelength of 590 nm, which was defined as Relative fluorescent unit (RFU) value (as explained in detail in the manuscript). Since the RFU values of the HGF cells cultivated onto polystyrene are statistically similar to the ones of the cells attached to the as-cast samples' surfaces ($p>0.05$, Fig. S3), as-cast samples were considered control samples for *in vitro* evaluation of cytocompatibility properties.

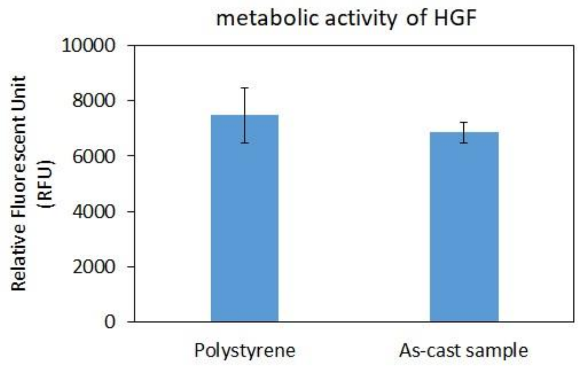

Figure S3. Comparison of the metabolic activity of the HGF cells cultivated onto the polystyrene with attached-cells to the as-cast samples' surfaces; $p>0.05$.

Supplementary Figure 4: Direct cytocompatibility evaluation

To evaluate the direct cytocompatibility assessment of TA-functionalized ribbons on HGF cells, the surfaces of the samples were seeded directly with cells; then, after 24 and 48 h of incubation, metabolic activity, viability, and morphology of the surface-attached cells were measured using the alamar blue assay, fluorescent Live/Dead assay, and scanning electron microscope (SEM), respectively and as-cast ribbons were considered control samples. The results are presented in Fig. S4 (a–c). The metabolic activity results taken after 24 and 48 hours demonstrated no statistically significant differences between the attached cells onto the surfaces of the TA-functionalized $Ti_{60}Zr_{20}Si_8Ge_7B_3Sn_2$ MG ribbons in comparison with as-cast samples ($p>0.05$, Fig. S4 (a)). Visual analysis of the attached HGF cells onto the specimens' surfaces, after 48 h of incubation, with the fluorescent Live/Dead stain revealed that mostly (>95%) HGF cells stained in green and low

amount of red-stained dead cells were observed, indicating the viability of the majority of the surface-attached cells (Fig. S4 (b)). These results were confirmed by SEM images demonstrating that the viable HGF cells proliferated properly on the surfaces of the both samples, as-cast and TA-functionalized ribbons (Fig. S4 (c)). These promising results show that TA-functionalized $Ti_{60}Zr_{20}Si_8Ge_7B_3Sn_2$ MG ribbons are safe for HGF cells' adhesion to their surfaces. As explained in detail in the manuscript, this cell-friendly behavior of the functionalized specimens can be due to the enhancement of protein adsorption in the early adhesion phase as well as in the subsequent deposition of extracellular matrix (ECM) because of the existence of pyrogallol groups exposed by the tannic acid.

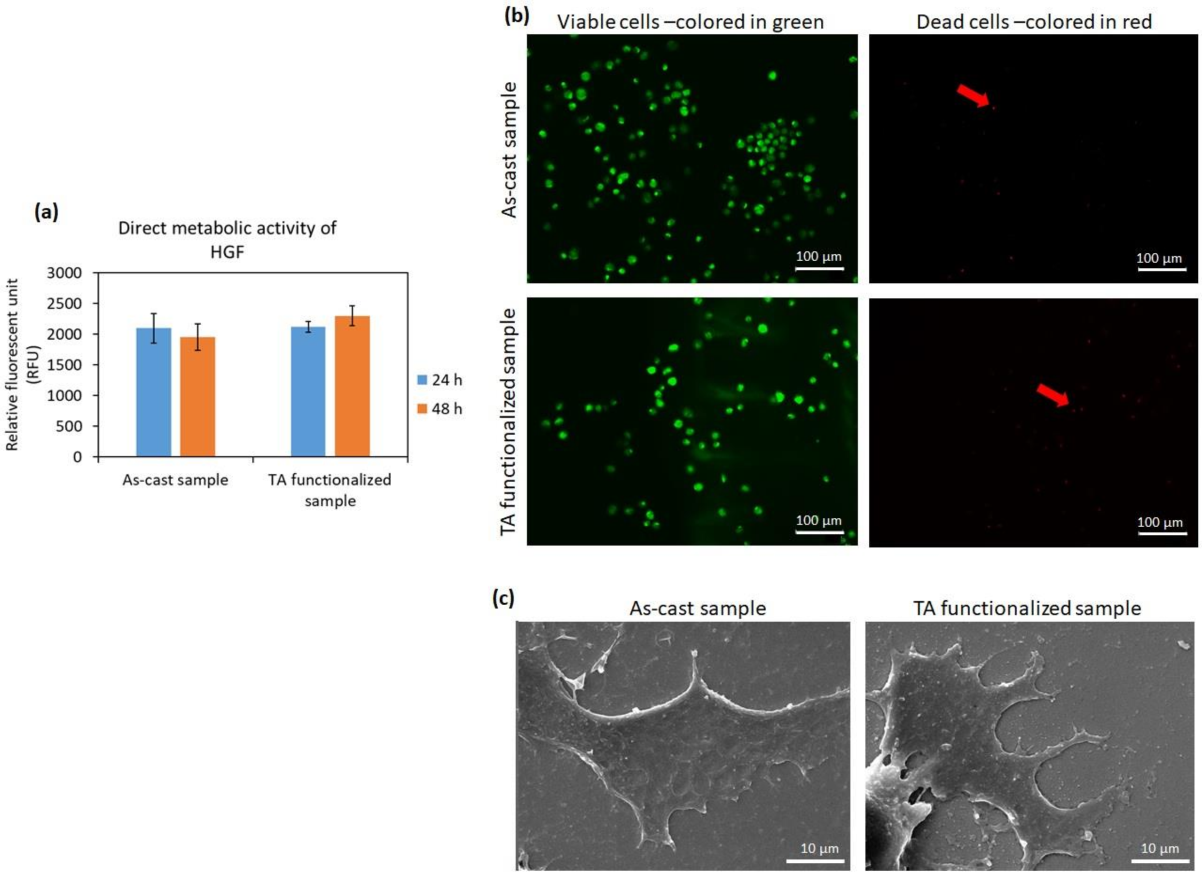

Figure S4. Direct cytocompatibility evaluation of $Ti_{60}Zr_{20}Si_8Ge_7B_3Sn_2$ MG ribbons on the HGF cells. **(a)** Metabolic activity of the cells after 24 and 48 h; **(b)** Fluorescent Live/dead assay after 48 h; left panel showed the viable cells which were colored in green and right panel indicates the dead cells in red color. Arrows indicate the dead cells. Scale bar = 100 µm; **(c)** SEM images taken after 48 h of incubation from the surface-attached cells. Scale bar = 10 µm.

Supplementary Table 1: The compositional analysis of all samples.

Sn2Ref	Atomic %	Sn2Ref Etch 300s	Atomic %	Tannic Acid	Atomic %	Tannic Acid Eth 300s	Atomic %
Zr3d5 ZrO2	3,57	Zr3d5 ZrO2	0,1	Zr3d5 ZrO2	3,06	Zr3d5 ZrO2	0,0
Zr3d5 metal	0,84	Zr3d5 metal	15,1	Zr3d5 metal	0,66	Zr3d5 metal	15,0
Ti2p3 TiO2	9,91	Ti2p3 TiO2	1,1	Ti2p3 TiO2	7,71	Ti2p3 TiO2	1,0
Ti2p3 metal	1,76	Ti2p3 metal	45,7	Ti2p3 metal	1,24	Ti2p3 metal	46,3
Sn3d5 metal	0,14	Sn3d5 metal	1,2	Sn3d5 metal	0,14	Sn3d5 metal	1,2
Sn3d5 SnO2	1,28	Sn3d5 SnO2	0,2	Sn3d5 SnO2	1,01	Sn3d5 SnO2	0,2
Si2p3 metal	0,9	Si2p3 metal	7,0	Si2p3 metal	0,87	Si2p3 metal	7,3
Si2p3 Si-O	2,83	Si2p3 Si-O	2,0	Si2p3 Si-O	2,02	Si2p3 Si-O	2,1
O1s Scan A	23,61	O1s Scan A	4,5	O1s Scan A	20,81	O1s Scan A	4,5
O1s Scan B	17,68	O1s Scan B	2,8	O1s Scan B	8,51	O1s Scan B	2,7
O1s Scan C	1,78	O1s Scan C	1,3	O1s Scan C	6,28	O1s Scan C	1,3
Ge2p3 Scan A	0,07	Ge2p3 Scan A	7,0	Ge2p3 Scan A	0,06	Ge2p3 Scan A	7,2
C1s sp3	23,09	C1s sp3	1,1	C1s sp3	32,82	C1s sp3	1,9
C1s C-O	6,93	C1s C-O	1,6	C1s C-O	10,63	C1s C-O	1,5
C1s carbide	0,45	C1s carbide	4,3	C1s carbide	0,33	C1s carbide	3,5
C1s OCO	3,97	C1s OCO	0,8	C1s OCO	3,22	C1s OCO	0,5
C1s carbid2	1,21	C1s carbid2	1,1	C1s carbid2	0,63	C1s carbid2	1,1
		B1s	3,3			B1s	2,7

Table S1. XPS elemental analysis of the investigated samples.
